# Structural variant detection in cancer genomes: computational challenges and perspectives for precision oncology

**DOI:** 10.1038/s41698-021-00155-6

**Published:** 2021-03-02

**Authors:** Ianthe A. E. M. van Belzen, Alexander Schönhuth, Patrick Kemmeren, Jayne Y. Hehir-Kwa

**Affiliations:** 1 Princess Máxima Center for Pediatric Oncology, Utrecht, The Netherlands; 2 Genome Data Science, Faculty of Technology, Bielefeld University, Bielefeld, Germany

**Keywords:** High-throughput screening, Genomic analysis

## Abstract

Cancer is generally characterized by acquired genomic aberrations in a broad spectrum of types and sizes, ranging from single nucleotide variants to structural variants (SVs). At least 30% of cancers have a known pathogenic SV used in diagnosis or treatment stratification. However, research into the role of SVs in cancer has been limited due to difficulties in detection. Biological and computational challenges confound SV detection in cancer samples, including intratumor heterogeneity, polyploidy, and distinguishing tumor-specific SVs from germline and somatic variants present in healthy cells. Classification of tumor-specific SVs is challenging due to inconsistencies in detected breakpoints, derived variant types and biological complexity of some rearrangements. Full-spectrum SV detection with high recall and precision requires integration of multiple algorithms and sequencing technologies to rescue variants that are difficult to resolve through individual methods. Here, we explore current strategies for integrating SV callsets and to enable the use of tumor-specific SVs in precision oncology.

## The importance of structural variant detection in cancer

**Fig. 1:**
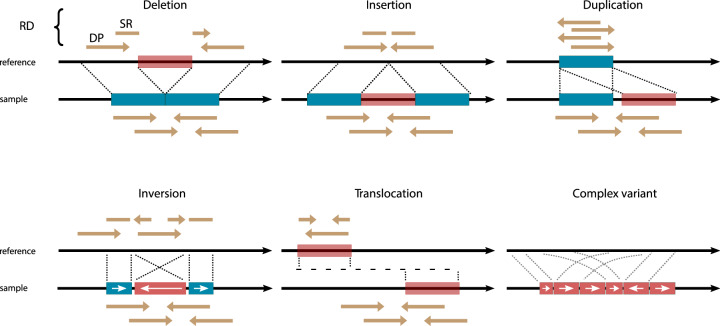
Major SV types and their characteristic read-alignment patterns. Alignment of paired-end sequencing reads to a reference genome is used to infer sites of discontinuity or breakpoints. Structural variants (SVs) are generally defined as larger than 50 base pairs and further classified in five major SV types: deletions, insertions of non-reference sequence or mobile elements, duplications, inversions and translocations. Clusters of breakpoints in a genomic region which cannot be classified are considered “complex SVs” and likely result from either progressive rearrangements or a major genomic disturbance. SVs (red blocks) are characterized by patterns in breakpoints and reads aligned to flanking reference sequences (blue blocks). The reads directly below the sample DNA strand represent the distance and orientation at which they are generated during sequencing. If the reads align differently than expected to the reference strand this is indicative of an SV. Changes in read depth (RD) or coverage indicate mostly larger duplications or deletions and are useful for detecting copy number variants (CNVs). Discordant pairs (DP) align to the reference at a different relative distance or orientation than expected. DPs are best suited for detecting large SVs such as inter-chromosomal translocations or inversions. Split reads (SR) span breakpoints and can only be partially aligned. SR can detect small variants with base-pair resolution, especially those smaller than the size of the read.

Genomic aberrations acquired in cancer genomes encompass a broad spectrum of types and sizes. These range from single nucleotide variants (SNVs) to larger structural variants (SVs) that impact genome organization (Fig. 1, Table 1)^1,2^. SVs are a major contributor to genomic variation, they affect more base pairs in the genome than SNVs^3^ and can have serious phenotypic impact^4,5^. Some SVs are known to drive carcinogenesis and SVs resulting in gene fusions were the first recurrent mutations observed in many pediatric cancers^6,7^. With at least 30% of cancer genomes affected by a pathogenic SV, detection of SVs is essential for both diagnosis and treatment stratification^6–11^. In addition, discovering new oncogenic SV driver events is beneficial for understanding cancer etiology. However, research into the role of SVs in cancer has been limited due to difficulties in their detection which has partially resulted from co-opting sequencing technologies designed for SNV detection.

**Table 1:** Glossary of key terms.

Breakpoint	The location at which a structural variant differs from the reference genome, and forms a novel junction between two previously unconnected segments.
Chimeric transcript	A transcript consisting of exons from two different genes, resulting from a genomic mutation or transcriptional process like intergenic splicing or read-through.
Complex rearrangement	Structural variant consisting of multiple breakpoints that can not be traced back to a basic type.
Differential analysis of tumor-normal data	Also known as “somatic analysis”. By using paired sequencing data, the aim is to classify detected variants as either tumor-specific or also occurring in the matching normal sample.
Discordant read pairs	Sequencing reads which have an abnormal insert size when mapped to the reference genome, either larger or smaller than expected, but also mapping to two different chromosomes.
Haplotyping/phasing variants	Determining if detected variants occur on the same homologous chromosome and potentially affect the same allele.
Long-read sequencing technologies	Single molecule sequencing technologies are actively developed by Pacific Biosciences and Oxford Nanopore Technologies. Reads are ~10 kb+ with a nucleotide accuracy of ~85% depending on the platform version and base calling algorithm (Table 3).
Polyploid	Cells which contain more than two chromosomes of each pair.
Read alignment patterns	Alignment of read pairs to a reference genome which behave differently than expected. Specific patterns can indicate a structural variant is present. Patterns include changes in read-depth, discordantly paired reads, split reads, soft-clipped reads and one-end mapped reads (Fig. 1).
Short-read sequencing technologies	Often used synonymously with sequencing-by-synthesis technology from Illumina. Generates paired-end reads of 150–250 bp with 99% nucleotide accuracy (Table 3).
Split reads	Sequencing reads that span breakpoints and therefore map to two locations (split reads) or can only be partially mapped to a single location (soft-clipped reads). Since the default aligner BWA-MEM soft-clips also split reads, they are often used synonymously.
Structural variant (SV)	Genomic variant larger than 50 bp in size. Five major SV types are distinguished: deletions, duplications, inversions, translocations and insertions of non-reference sequence or mobile elements.
Tumor purity	The proportion of cancer cells within a tumor sample.
Variant allele frequency	The relative abundance of a variant allele versus the unchanged reference allele based on read support.

Advances in sequencing technologies have increased the number of SVs identified per genome from ~2, 1–2, 5k in the 1000 genomes project to more than 27k in recent multi-platform sequencing efforts^3,4,12^. Specifically for the cancer genomics community, recent contributions of the Pan-Cancer Analysis of Whole Genomes (PCAWG) Consortium have provided an extensive resource of paired tumor-normal genomes^13^. The insights obtained from multi-platform analyses also highlight current SV blindspots in cancer variant databases like COSMIC. Despite technological innovations, confident SV detection in cancer genomes remains challenging due to biological factors including contamination from healthy tissue, intratumor heterogeneity and polyploidy. Identification of variants acquired in tumor cells requires discerning tumor-specific somatic SVs (TSSVs) from variants in the germline and mosaic variants present in unaffected cells^14^. This is often done by differential analysis between paired tumor-normal samples^15^. The classification of SVs as tumor-specific or normal is confounded by inconsistencies in detected breakpoints and derived variant types, as well as the biological complexity of some rearrangements.

Confident SV detection and subsequent classification of variants as either germline, tumor-specific or mosaic variation in healthy tissue is not only important for diagnostics and cancer etiology but also for research into cancer predisposition and genetic interactions. In addition, the genetic context of somatic variants and interplay with germline variants may influence their tumorigenic potential^16^. Here, we focus on the detection of TSSVs from paired tumor-normal WGS data. First, we explore current approaches for SV detection and their integration, whilst accounting for challenges specific to cancer samples. Second, we address different approaches aimed at distinguishing TSSVs from normal SVs. Third, we highlight the impact that long-read sequencing can have on somatic SV detection. Last, we explore how orthogonal sequencing technologies can be combined to improve TSSV detection.

## Detection of somatic SVs in short-read WGS data

SVs can be detected using short-read sequencing data based on patterns in aligned reads (Fig. 1). These reads are sequenced as paired ends of 150–250 bp long. Changes in read-depth (RD) are used to derive copy-number variants (CNVs). Discordant read-pairs (DP) that align with an abnormal distance and/or orientation to the reference genome are suited for detecting large SVs. Split or soft-clipped reads (SR) are partially mapped reads and can indicate breakpoints with base-pair resolution^17^. Both the alignment method and reference genome used, influence the performance of SV detection algorithms^17,18^. BWA-MEM is predominantly used for alignment prior to SV detection, as it provides secondary alignments to reads mapping to multiple locations rather than placing the reads randomly^19,20^. However, alignment uncertainty is inherent to short-read sequencing data. In parallel, the reference genome continues to evolve, resulting in improved alignments and fewer false-positive variants in studies which adopted GRCh38 (hg38) compared to GRCh37 (hg19)^8,21–23^.

### Combinatorial algorithms integrate multiple read-alignment patterns

**Fig. 2:**
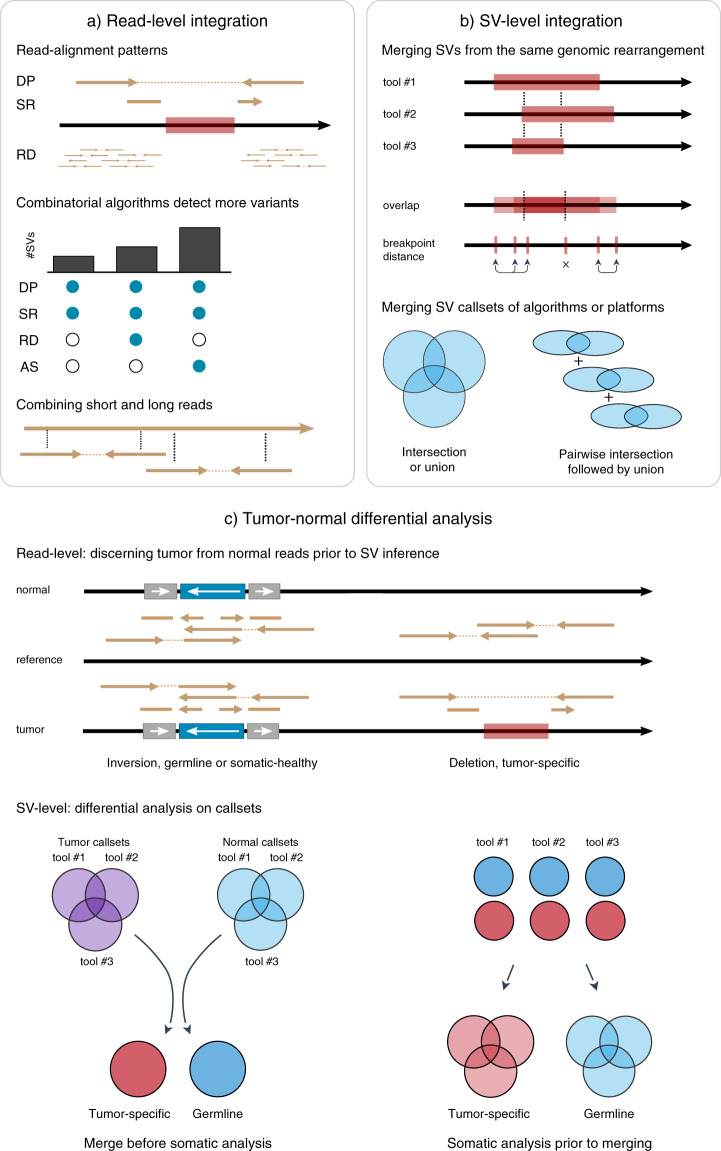
Data integration to improve tumor-specific SV detection. **a** Alignment of sequencing data against a reference is used to infer SVs by detecting aberrant patterns of read-alignment: discordant pairs (DP), split reads (SR), read depth (RD) and (local) assembly (top, see also Fig. 1). Algorithms that combine multiple read-alignment patterns can resolve more SVs (middle). Likewise, read-level integration of technologies can aid SV detection, i.e., combining short and long reads (bottom). **b** Comparison of SV callsets requires merging variants from the same genomic rearrangement based on e.g., reciprocal overlap or breakpoint distance (top). These merging approaches can yield different outcomes as shown by how only a small segment of the deletion overlaps between tools and not all breakpoints could be matched. Intersection of callsets identifies the SVs with support from multiple algorithms or technologies. Alternatively, sensitivity can be increased by taking the union of callsets or their pairwise intersections (bottom). **c** Identification of tumor-specific SVs (red) requires tumor-normal differential analysis of reads or events. A tumor sample (purple) is expected to contain tumor-specific variants (red, bottom stand), as well as germline variants (blue, top strand). Tumor/normal reads can be distinguished prior to SV inference or afterwards by comparison of the variants or breakpoints as in **b**. If multiple SV tools are used, differential analysis can be done after merging tumor and normal callsets (bottom left) or first by using each algorithm’s somatic filtering feature (bottom right).

The latest generation of SV detection algorithms that combine multiple read-alignment patterns can detect SVs across a broad range of types and sizes. At present, many different strategies and methods exist (Table 2). How these combinatorial algorithms integrate read-alignment patterns influences their ability to detect specific variant classes (Fig. 2A)^24,25^. As a result, no single algorithm performs best across the full spectrum of SVs, implying that integration of multiple algorithms is beneficial^25^. Although most studies comparing SV algorithms focus on germline SVs, these findings were recently also confirmed for somatic SV detection^26^. The methodology used by DELLY, LUMPY, Manta, SvABA, and GRIDSS for detecting SVs (Box 1) achieves high performance in detecting both germline and somatic SVs^25,26^.

**Table 2:** SV detection algorithms.

Tool^1^	Platform	Method	Reference	Used in study
DELLY	IL	DP, SR	^34^	^8,13,89,93^
LUMPY	IL	DP, SR	^35^	^8,81^
Manta	IL	DP, SR, AS/l	^36^	^89,94^
GRIDSS	IL	DP, SR, AS/l	^37,39^	
SvABA	IL, 10×	DP, SR, AS/l	^38^	^13,83^
Varlociraptor	IL	Post-processing	^31^	
Lancet	IL		^40^	
GROC-SVS	10×		^95^	^81,83^
Longranger	10×		^96^	^81,83^

Long read tools	Platform	Method	Reference	Cited by/remarks
HySA	PacBio and IL	Assembly and alignment	^76^	Hybrid assembly of IL and PacBio reads
SVIM	ONT, PacBio	Alignment	^69^	^67,93,97^
Sniffles	ONT, PacBio	Alignment	^55^	^25,32,56,67,89,93,94,97,98^
pbhoney	PacBio	Alignment	^99^	^25,76^
pbsv	PacBio	Alignment	^100^	^25,97^
NanoSV	ONT	Alignment	^98^	^32,56,93^
Picky	ONT	Alignment	^56^	^32,93^
NanoVar	ONT, PacBio	Alignment	^93^	Applied to a leukemia sample
cuteSV	ONT, PacBio	Alignment	^97^	
nanomonsv	ONT	Alignment	^68^	Detects tumor-specific SVs

^1^Inclusion criteria: published tool focussed on tumor-specific SV detection in cancer from tumor-normal paired WGS data, used in key studies addressed in this review. Long-read alignment based SV detection tools that are commonly used are included regardless of their ability to detect tumor-specific SVs.

Box 1: Integration of read-alignment patterns by combinatorial algorithms Integration of read-alignment patterns by SV detection algorithms influence which SVs can be confidently detected. DELLY, LUMPY, GRIDSS, Manta, and SvABA are state-of-the-art algorithms and have amongst the best performance for germline SV detection^25^. They can detect all the major SV types at base-pair resolution using SR or assembly and also perform somatic classification.

DELLY uses DP and SR in a stepwise manner to detect ~200 bp–5 kbp SVs^34^. Since DELLY analyses SV types separately, it can detect nested SVs and infer complex events which is useful for somatic SV detection. LUMPY has a probabilistic model that combines parallel analyses of DP and SR such that both contribute independently to the detection of breakpoints^35^. Overlapping breakpoints are clustered to identify SVs, except for insertions. GRIDSS can detect SVs and indels regardless of size using a combination of assembly, SR and DP-support^37^. Break-end contigs spanning SV breakpoints are assembled from SR, DP, one-end anchored, gapped, and unmapped reads. Variants are inferred with a probabilistic model combining evidence from realignment of these break-end contigs, SR and DP. GRIDSS can rescue un/misaligned reads, detect novel non-reference sequence insertions, and resolve micro-homology surrounding breakpoints. Manta uses a graph-based approach to generate candidate SVs from DP, SR and gapped reads, followed by local assembly and realignment of contigs to the genome. SVs are scored by a model that integrates evidence from discordant reads and the assembly. SvABA performs genome-wide local assembly in 25 kb windows based on SR, DP, gapped, and unmapped reads^38^. Variants are inferred from alignment of contigs to the reference and subsequently scored by realignment of reads to the contigs.

Despite their differences in approach, for overlapping/shared SVs these tools agree on breakpoints within ~2 bp based on simulations in optimal detection conditions^26^.

### SV-level integration of multiple algorithms improves precision

Since the optimal detection algorithm differs between SV type and size range, full-spectrum SV detection with high recall and precision currently requires multiple algorithms^25,27^. The optimal method to combine the resulting callsets remains a largely unanswered question and a variety of tools and in-house pipelines are currently used^4,13,25,28^. To compare and combine SV callsets, variants from the same genomic rearrangement need to be merged first, this is complicated by diversity in breakpoint resolution and SV typing (Fig. 2B). The recent review by Ho et al. addresses different “ensemble” integration approaches currently in use in germline SV research^4^. In general, simple integration strategies use (reciprocal) overlap or breakpoint distance to merge SVs whilst more complex solutions combine this with read-evidence integration, local assembly or machine learning^29–32^.

After overlapping variants are merged, integration of SV callsets from multiple algorithms can either be performed by taking the union or intersection (Fig. 2B). Since achieving high precision takes priority in most cancer research and clinical applications, an intersection strategy is often preferred but reduces recall. The precision/recall trade-off can be optimized by carefully selecting which tools to intersect^25^ and by taking the union of pairwise intersections^26^.

## Distinguishing somatic from germline SVs

TSSV detection aims to identify variants that uniquely occur in a patient’s tumor cells. Typically paired tumor-normal samples are used to classify SVs as either germline, mosaic-normal or tumor-specific variants^15^. Detection of TSSVs is a two-step process that involves the detection of SVs in both samples, followed by differential analysis of the callsets (Fig. 2C). Also, cancer genomes can have highly complex rearrangements. Alternatively, if patient-derived healthy material is not available, SVs can be filtered using a panel-of-normals. A sufficiently large panel-of-normals can provide more statistical power for filtering recurrent germline variants, but is less effective than a patient-derived normal sample when filtering rare or private germline variants^4^. Also, strictly filtering out regions with germline CNVs excludes potentially interesting genomic regions from SV analysis, which are susceptible to rearrangements because of their architecture^33^.

### Tools for somatic SV detection in WGS data

Somatic SV detection algorithms differ in their approach to identify TSSVs from paired tumor-normal samples and as a result can classify the same event differently^26^. Despite their differences, DELLY, LUMPY, SvABA, Manta, and GRIDSS have successfully been used to report somatic SVs in various studies^34–37^. DELLY and LUMPY use ad hoc filtering whereby SVs supported by at least one read from the normal sample are removed from the tumor SV callset^34,35^, which is highly sensitive contamination. In contrast, Manta uses a probabilistic scoring system for somatic SVs integrating evidence from tumor and normal reads^36^. SvABA uses both the tumor and normal data during assembly before distinguishing somatic variants^38^. GRIDSS has yet another approach and applies extensive rule-based filtering to both single break-ends and breakpoints^37,39^.

Specialized somatic SV detection tools such as Lancet and Varlociraptor account for challenges specific to the identification of TSSVs (Box 2)^31,40^. The first challenge in comparing tumor and normal SV callsets are differences in SV breakpoints and types, analogous to the issues with overlapping SV callsets of different algorithms^25^. Second, somatic SVs are often complex which can be problematic for algorithms that are not equipped to resolve these complex SV signatures and instead infer (false-positive) small indels^41^. As an alternative to ad-hoc filtering of SV callsets, Varlociraptor and Lancet, respectively, compare breakpoints and aberrant reads between tumor-normal samples at an earlier stage of the analysis (Fig. 2C). Specifically, Varlociraptor compares the statistical support for an altered reference with simulated variant versus an unadjusted reference (Box 2)^31^. Using read-level or breakpoint-level comparison can account for the subsequent mutations at germline variant locations, as these mutations may convolute somatic-germline comparisons. Third, issues inherent to analyzing tumor samples such as contamination, polyploidy, and heterogeneity are accounted for by Varlociraptor and Lancet (Box 2).

Box 2: SV detection algorithms specialized in differential analysis Lancet and Varlociraptor address challenges specific to tumor-normal analysis, e.g., contamination, polyploidy, intratumor heterogeneity (subclonality) and thus aid in identification of tumor-specific SVs.

Lancet is specialized in the detection of somatic SNVs, insertions (<200 bp) and deletions (<400 bp) from short-read WGS data using local (micro-)assembly and re-alignment to the reference^40^. By using a graph-based approach, Lancet can resolve haplotypes and use the origin of supporting reads to distinguish TSSVs from germline variants. Sample contamination can be accounted for by adjusting the number of allowed supporting normal-reads. Lancet can detect rare variants (>5% AF) in a virtual tumor whilst preventing false-positives in short-tandem repeat regions, achieving higher precision than other algorithms but at cost of sensitivity.

Varlociraptor is a post-processing tool which uses a Bayesian framework to differentiate between somatic and germline breakpoints by calculating false discovery rate (FDR) values from unfiltered callsets^31^. During FDR calculation it quantifies uncertainties due to ambiguous read alignments, how reads support SVs (typing uncertainty), gap-placement bias and strand bias^30,31^. This is done by simulating the variant into the reference, re-aligning reads and comparing the statistical support for the adjusted versus unadjusted reference. Challenges specific to tumor samples are taken into account, as additional uncertainties e.g., mosaic-normal variants, contamination, intratumor heterogeneity and aneuploidy. By doing so, it is able to control the FDR of SNVs and small insertions/deletions (30–250 bp) and achieves better precision/recall on callsets of DELLY, Manta, and Lancet compared to the filtering of the tools themselves^31^.

## Challenges for accurate SV detection in cancer genomes

The analysis of tumor-normal paired samples is confounded by challenges inherent to cancer samples, including polyploidy, heterogeneity and contamination^17^. First, potential aneuploidy of tumor cells complicates haplotype reconstruction and phasing reads^12,42^. Second, intratumor heterogeneity can result in multiple subclonal variants which have low allele frequency (AF) and few supporting reads, making them difficult to detect. Third, contamination of the tumor sample with healthy material and vice versa complicates differential analysis between paired samples due to mislabelled reads. This can result in algorithms falsely discarding somatic variants with one or more supporting reads from the control sample. Adjusting the filtering threshold based on an estimated contamination fraction is a balance between precision and sensitivity for detecting low-AF variants.

The detection of rare TSSVs is limited by sequencing depth and AF. In practice, a minimum of 20% AF is required for reliable variant detection from tumor-normal pairs^26,31^. Increasing sequencing depth to 75x-90x for tumor samples improves the sensitivity of detection, especially for variants below 20% AF, whilst maintaining precision^26^. In addition, interpretation of TSSV allele frequencies is not straightforward since they can reflect intratumor heterogeneity and/or multiple alleles within a polyploid tumor genome. Note that the SV type should be considered during AF interpretation^43^. For diploid normal cells, variants are expected to have an AF of 0%, 50%, 100%, or 33% in case of a heterozygous duplication. However, mosaic-normal variants can occur at varying AF and be difficult to distinguish from TSSVs^14^. Computational modeling with AF can provide insight into intratumor heterogeneity and clonal architecture, both of which are important for therapeutic resistance and relapse^44^. The majority of SV tools operate under a diploid genome assumption. A multitude of tools independently quantify purity and ploidy of tumor samples however benchmarking studies show little consensus^39,45^. These tools can rely solely on CNV deletion events to model the cell purity and ploidy, and/or incorporate heterozygous known SNPs into their probabilistic models. At present, only SVclone uses SVs to estimate intra-tumor heterogeneity due to the complexities of calculating variant AF for SVs^43^.

### Computational challenges of complex variant detection

Genomic instability in cancer genomes results in more breakpoints and more complex SVs compared to germline variation^46^. Complex SVs are characterized by signatures of many breakpoints clustering together and are hypothesized to be caused by a single catastrophic process followed by repair or progressive rearrangements^47^. The presence of breakpoint clusters complicates the inference of the underlying genomic rearrangements and therefore also the identification of tumor-specific events. Alternatively, when breakpoint clusters confound confident SV calling, breakpoint-level differential analysis can be used to identify tumor-specific events. In addition, unsupervised clustering can discern complex from simple SVs and help to study both events more accurately^41^.

### Technical limitations of short-read WGS influence SV detection

The detection of SVs is also influenced by technical limitations of the sequencing platform; most notably genome coverage bias and alignment uncertainty. Illumina (IL) is currently the most commonly used short-read sequencing platform since it’s relatively affordable, fast and has a high nucleotide accuracy (>99%)^48^. However, IL sequencing has inherent biases in genome coverage with regions that have a high, or low GC content (<10% and >85% GC) or long homopolymers^49^. Although PCR-free library preparation does reduce GC biases it does require a large amount of input DNA (Table 3)^49^.

**Table 3:** Comparison of long-read and short-read sequencing technologies.

	Illumina	10× linked-read	Short-read RNA-seq	
Avg read length	2× 150–250 bp	IL platform dependent	2× 75 bp	
Max read length	2× 250 bp	(~100 kb span)	2× 100 bp	
Accuracy per nucleotide^1^	>99%	(see Illumina)	(see Illumina)	
Error bias	Substitutions in high/low GC regions	(see Illumina)	(see Illumina)	
Coverage bias	Low coverage of high/low GC regions. Mapping issues with highly homologous regions	(see Illumina)	Illumina biases and additional: ligation bias due to the reverse transcriptase enzyme^101^, protocol differences poly-A only versus ribodepletion	
Accuracy after error correction^2^	N/A	N/A	N/A	
Sample requirements PCR-free^3^	1–2 μg/(default > 500 ng)	IL + controller chip 1 ng	0.1–1 μg	
Low-throughput sample requirements	100 ng^3^	–	25 ng	
Base modifications	Bisulfite sequencing required	–	–	

	Nanopore	PacBio	BNG	Hi–C
Avg read length	15–20 kb	10–15 kb	~100 kb resolution, variants >500 bp	~1 kb–1 Mb resolution^102^
Max read length	>800 kb^103,104^	>60 kb^103–105^		
Accuracy per nucleotide^1^	60–85%^103,104,106^	>85%^105^	no base pair resolution	no base pair resolution
Error bias	Small indels, mostly deletions^49,107^	Small indels, mostly insertions^49,107^	N/A	N/A
Coverage bias	Truncation of homopolymers and low-complexity regions^103^	Homopolymers	Fragile sites, nick enzyme-dependent^108^	Biases depend on protocol used: restriction enzymes, PCR, and IL seq^102,109^
Accuracy after error correction^2^	After 1D^2^ 97% After hybrid correction: >99%	After CCS: 95–99%^60^.	N/A	N/A
Sample requirements^3^	1 μg–400 ng HMW 0.4–1 μg HMW	10 μg HMW	0.3–0.9 μg HMW	1–10 million cells^102,109^
Low-throughput sample requirements	10–100 ng	400–800 ng	300 ng	1000 cells^110^
Base modifications	Theoretically all	Methylation, Mostly bacterial	–	–

Comparison of Pacific Biosciences (PacBio), Oxford Nanopore Technologies (ONT), Illumina (IL), 10× Genomics linked-read sequencing on the Illumina platform (10×), RNA sequencing on the Illumina platform (RNA-seq), BioNano Genomics (BNG) and the genome-wide chromatin conformation capture protocol Hi–C (Hi–C). Many characteristics of 10× and RNA-seq are shared with IL, since the same sequencing platform is used. Also note that Hi–C protocols are under active development, they vary in biases, sample requirements and use of IL sequencing^102,109,110^. Unreferenced values are derived from the manufacturer’s websites last accessed in October 2020 [Oxford Nanopore Technologies (https://nanoporetech.com/), PacBio (https://www.pacb.com/), Illumina (https://www.illumina.com/), 10x Genomics (https://www.10xgenomics.com/), Illumina Stranded mRNA Prep (https://www.illumina.com/products/by-type/sequencing-kits/library-prep-kits/stranded-mrna-prep.html), Bionano Genomics (https://bionanogenomics.com/)].

^1^Reported accuracy for PacBio and ONT strongly depends on the sequencing platform version and polishing steps. Using regular single-pass sequencing without self-correction, both PacBio and ONT theoretically have per-nucleotide error rates of ~15%^103–105^ but previous versions of the MinION up to ~40%^106^.

^2^The latest ONT and PacBio technologies attain >99% accuracy for de novo human assemblies. PacBio achieves >99.8% accuracy using circular consensus sequencing (CSS) where the same read is sequenced many times and averaged, although this limits read length to ~13 kb^60^. ONT reports >99% after polishing with short reads (hybrid correction) which is necessary due to truncation of homopolymers and low-complexity regions^103^. ONT 1D^2^ technology sequences both DNA strands and uses consensus to attain >97% whilst maintaining read lengths, although only ~60% of the molecules can be sequenced using this approach [Oxford Nanopore Technologies (https://nanoporetech.com/)].

^3^Sample requirements as listed by the manufacturer and dependent on the library preparation method used, e.g., insert size and use of PCR, as well as the exact version of the machine. High molecular weight (HMW) DNA is required to attain long read lengths, but the read length of PacBio is limited by the polymerase and for ONT by the length of the DNA molecules hence it can report ultra-long reads >800 kb^103^. The minimum sample amount of 10 ng listed by ONT is likely insufficient for a human genome. Whilst for IL in practice smaller amounts e.g., 50 ng are used as low-throughput minimum.

The detection of SVs relies on identifying aberrant read alignment patterns (Fig. 1). Reads derived from highly homologous regions, such as pseudogenes and segmental duplications, are often not long enough to uniquely map to the reference genome^50^. Yet repeat-rich regions comprise about half of the human genome and are vulnerable to SVs due to homologous recombination errors and replication slippage^33,51^. Depending on the alignment algorithm, uncertainty usually results in either random placement of reads or multi-mapping to all possible locations^52^. Multi-mapping, for example as done by BWA-MEM, causes unequal genome coverage altering the signal-to-noise ratio^52^. Hence, alignment uncertainty is problematic for accurate SV detection and should be addressed with a sound statistical model^30,31,52^. Current estimates suggest ~55 Mb of GRCh38 are “dark regions” inaccessible to IL sequencing due to alignment ambiguity (i.e., repeat-rich regions) or the sequencing chemistry (i.e., GC content)^53^. The over 4000 affected gene bodies^53^ also include disease-related genes, such as the TERT promoter which was found to be mutated in 9% of tumors in the PCAWG study but mutations can be missed due to its high GC content^13^.

## Impact of long-read sequencing

Single-molecule long-read sequencing technologies by Pacific Biosciences (PacBio) and Oxford Nanopore Technologies (ONT) are valuable for SV detection^54^. PacBio and ONT generate reads of ~10+ kb versus ~250 bp from IL; the longer reads reduce alignment ambiguity and do not have a GC bias, resulting in improved coverage of “dark” regions in the genome^55^. In addition, long reads allow for haplotype phasing of variants and *de novo* assembly of complex rearrangements^56^. For example, sequencing lung cancer cell lines with PromethION detected both known cancer-driver SNVs and revealed large previously unknown genomic rearrangements, including an 8 Mb amplification of *MYC*^57^. Similarly, direct comparison of a PacBio assembly with IL sequencing shows ~2.5× more uniquely identified SVs (~48k and ~20k, respectively), in particular more inversions and 50 bp–2 kb insertions/deletions located in repeat-rich regions^12^.

### Limitations of long-read sequencing

The disadvantages of PacBio and ONT platforms include costs and sample requirements, which are substantial compared to IL sequencing and can be problematic for tumor samples (Table 3)^55^. In addition, they have a lower nucleotide accuracy of ~85% for single molecule sequencing and up to 99% using consensus sequencing of the same DNA molecule^58–61^. Continuous improvements in algorithms for base calling and error correction have increased the accuracy of these platforms^58,59^. Since low nucleotide accuracy can impede read-alignment, error correction potentially improves SV detection by increasing the fraction of aligned reads^62^. However, error-correction strategies come with trade-offs for SV detection. Long reads can be aligned to each other as a self-correction strategy when sufficient coverage (~50×) is available^55^. However, haplotyping information is lost as a result of using the consensus of reads with mixed molecular origin. This makes the consensus sequence unsuitable for variant phasing or for studying intra-tumor heterogeneity or polyploidy. Alternatively, short reads can be used for error correction by aligning them to the long reads, but this approach only improves accuracy of genomic regions accessible to IL sequencing^55,61^.

### Long-read data requires specialized algorithms

Long-read SV detection algorithms are either based on de novo assembly or read-alignment to a reference genome. Assembly-based strategies have a higher sensitivity for detecting non-template insertions and homozygous SVs. During assembly, contigs are compared to the reference genome and can provide more evidence than individual reads^32,55^. However, variant calling using alignment requires less coverage than assembly (~20× versus ~50×) and statistical significance when identifying SVs is achieved relatively easily due to the low alignment uncertainty of long reads^32,50,55^. Compared to assembly methods, alignment-based approaches are more suited to identify heterozygous SVs and more robust to amplifications in highly homologous regions such as low-complexity regions^12,55^. Within clinical applications, often insufficient resources are available to perform long-read sequencing of tumor-normal pairs to depths required for de novo assembly (Table 3). Therefore, we focus on using alignment-based strategies (Table 2).

Alignment of long reads differs from short reads due to the increase in base pairs to align and different errors profiles^55^. Although BWA-MEM offers support for long reads, it often infers many small gaps during alignment and misses large indels^63,64^. Specialized long-read alignment algorithms have been developed to overcome these issues. In contrast to short-read data, there is no best practise for which aligner should be used when performing SV detection^63–66^. Preliminary comparisons suggest that NGMLR and minimap2 perform well and both algorithms are designed to handle the higher error rates and adjust for the 1 bp indels in long-reads^12^.

### Alignment-based SV detection algorithms for long-read data

Currently, many tools are actively developed to detect SVs from alignment of ONT and PacBio data (Table 2). However, studies comparing long-read SV detection tools have been scarce and predominantly show the limitations of available truth sets by identifying many novel variants^12,67^. At present only nanomonsv reports somatic SVs from long-read data^68^. The commonly used tools SVIM and Sniffles have shown good precision and sensitivity in multiple performance assessments^63,67,69^. They were among the first to process both ONT and PacBio data despite their different error profiles and have been followed by additional tools like NanoVar and CuteSV (Table 2). Similar to short-read SV detection tools, long-read tools combine multiple read-alignment patterns to detect SVs. They infer patterns similar to split reads and discordant pairs using intra-alignment and inter-alignment signatures, despite long reads not being paired-end. Similar to short-read tools, using a consensus callset created by intersecting multiple long-read SV detection algorithms can increase precision^32,67^. Alternatively, machine learning approaches can attain greater improvements in precision and sensitivity than ad hoc intersection, given a truth set is available for training^32^.

## Multi-platform data integration to improve detection of somatic SVs in cancer

Limitations in both short-read and long-read WGS can potentially be overcome by using a multi-platform approach and as such improve the identification of TSSVs. Integration can improve both precision and sensitivity by combining read-alignment patterns (Fig. 2A) and integrating SV callsets from multiple algorithms or technologies (Fig. 2B).

### Gene fusion detection by combined analysis of RNA and WGS

Integration of genomic and transcriptomic data can further improve variant detection and provide insight into the phenotypic effect of SVs; specifically resolving gene fusions, splice variants and linking SVs to altered gene expression^70^. RNA sequencing of tumor samples offers unique advantages such as tissue specificity and time specificity, but obtaining high-quality RNA can be problematic. In addition, sufficient expression is necessary to detect events, which may impede detection of low AF variants.

RNA-seq is especially suitable for detecting gene fusion events through their chimeric transcripts. Gene fusions have high clinical relevance since they are often cancer drivers and otherwise occur rarely in the general population^6,70^. Specialized gene fusion algorithms predict gene fusions from chimeric transcripts by using read-alignment patterns such as SR crossing exonic junctions and DP mapping to both gene partners^71^. However, these algorithms can suffer from a high false positive rate which requires extensive filtering^72^. Chimeric transcripts can occur without genomic rearrangement, for example through intergenic splicing (*trans*-splicing and *cis*-splicing) or transcriptional slippage on short homologous sequences^73^. Since these chimeric transcripts are also present in healthy cells, this advocates for tissue matched RNA-seq of paired tumor-normal samples to allow the identification of tumor-specific events.

Combining RNA-seq with WGS data could resolve specificity issues and improve gene fusion detection. By itself, WGS can detect gene fusions, but not the occurrence of functional transcripts. Although sometimes used for validation purposes^74^, there are no established algorithms which integrate WGS and RNA-seq such that they both contribute to detection. The advantages of combining WGS, RNA-seq and exome sequencing has been demonstrated for detecting SVs in heterogeneous pediatric cancers^75^. Similarly, joint analysis of RNA-seq and short-read WGS in the PCAWG study identified the underlying SV for 82% of gene fusions. The remaining fusions were either the result of RNA-only alterations such as transcriptional read-through or underdetection of SVs^5^.

### Integration of short-read and long-read WGS

Short-read and long-read data can complement each platform’s strengths and overcome individual limitations^12^. Combining SV callsets after detection can increase sensitivity and requiring orthogonal support for variants across platforms can increase their confidence. However, the union or intersection of callsets is still affected by platform-specific technical biases. Read-level integration can overcome some of these issues as illustrated by error correction approaches which use IL reads to improve the accuracy of PacBio/ONT reads^55^. Likewise, hybrid assembly of short and long reads benefits from their respective high accuracy and scaffolding properties. Localized hybrid assembly tailored to SV detection as implemented by HySA shows that problematic SVs can be detected that have too little support in either PacBio or IL^76^. However, HySA cannot infer somatic SVs and some variants were missed due to few supporting aberrant IL reads and PacBio alignment issues. Hybrid assembly can also reduce coverage requirements for de novo assembly^77^.

As an alternative to long-read technologies, linked-read sequencing from 10× Genomics (10×) performs well for haplotype construction and variant phasing^12^. A read-barcode is added during library preparation to trace the molecule of origin at costs similar to IL sequencing^78^ (Table 3). In addition, 10× can report variants in repeat-rich regions not accessible by standard short-read IL sequencing^79,80^. Integration of short-read WGS and 10× enabled chromosome-scale haplotyping and phasing of detected variants of the polyploid cancer cell line HepG2^81,82^. Variant phasing can help to gain biological insights, as shown for associated regulatory and coding mutations in treatment-resistant prostate cancer^83^ and identification of SVs as potential cancer drivers by altering *cis*-regulation of genes^84^.

### Discovery of large, complex variants by chromatin assays

Combining sequencing data with technologies that provide insight into genomic organization can elucidatie large complex rearrangements. Technologies such as Bionano Genomics (BNG) and Hi–C have shown limitations of SV detection using sequencing. The combination of short-read WGS, BNG, and Hi–C on a cancer cell line showed most of the large (>1 Mb) intra-chromosomal and inter-chromosomal SV events were uniquely detected by a single technology with only ~20–35% validated by multiple platforms^8^. Each platform has its own scope of variant detection. Short-read WGS detected the largest number of variants across a broad range, whilst BNG and Hi–C lack base-pair resolution but can detect >1 kb deletions in repeat rich regions unlike short-read WGS^8^. BNG has promising diagnostic applications as it can confidently detect large variants with low input requirements (Table 3). Also, BNG had full concordance with standard diagnostic assays in pediatric ALL and identified additional variants^85^.

### Incorporating pre-existing technologies in ongoing studies

Continuous technological improvements provide exciting new data and SV discoveries, but this does not make existing datasets obsolete. The phenotypic effect of CNVs is often better understood than for SVs and established technologies have had more opportunity to collect samples, including rare cancer types. Currently many samples are available in repositories that profile genomic imbalances either via SNV array or exome sequencing technologies^13,86^. Challenges in integrating these datasets result from differences between technologies, such as breakpoint resolution and platform-specific biases, and systematic solutions are rare^87^. The widely varying detection resolution of different technologies invalidates callset intersection strategies, as smaller events are below the detection limits for lower resolution arrays, and exome sequencing is limited to events involving multiple exons. The absence of an event in a callset should not be considered proof that the event does not exist. Gene-centric approaches based on unions seem the most applicable. Although integration of pre-existing datasets assayed with different technologies with recently acquired datasets provides a complex computational challenge and is often ignored, it is likely to be an ongoing issue as technologies and platforms continue to evolve.

### Challenges in using sequencing for precision oncology

In clinical practice, next-generation sequencing (NGS) is increasingly used to replace targeted assays subject to budgetary and sample requirements. NGS can simultaneously detect different variant types and discover new biomarkers, and is more cost-effective than a series of single-gene assays. Although turn-around times are often longer, sensitivity and precision are maintained^88^ provided sufficient sequencing depth is achieved^26,31^. As a result, NGS makes pan-cancer biomarker testing feasible, leading to the approval of drugs based on molecular alterations shared by different cancer types like the use of TRK inhibitors for all solid tumors with a NTRK fusion^88^. However, the distribution of NGS data over multiple repositories and lack of data harmonization complicates clinical decision-making and prevents precision medicine from reaching its full potential.

Variant interpretation is a major challenge in precision oncology often done by expert panels such as interdisciplinary molecular tumor boards^88^. Despite its challenges, integration of multi-omics data is increasingly being used to improve variant interpretation and increase the number of identified drivers or actionable targets^5,88,89^. However, standards on variant interpretation and prioritization are still emerging^90^. As a result, there is low concordance between the recommendations of different molecular tumor boards when given identical case studies, especially for complex genomic alterations^90^.

Recent initiatives have attempted to resolve this need for standardization in variant assessment and clinical decision through the Molecular Tumor Board Portal^91^ and Somatic Working Group of the Clinical Genome^92^. Both harmonize different variant repositories, curated knowledge bases and computational predictions to acquire insights into variant-gene-drug-disease relationships with the focus on clinical use Although extremely valuable, these efforts focus only on SNVs and to a limited extent gene fusions. Similar initiatives for SVs and complex genomic alterations are currently lacking. Largely due to tumor-specific SVs not yet commonly being used as molecular targets or biomarkers to guide patient-specific treatment. We anticipate that improved confidence of TSSV detection will enable the subsequent research necessary for the use of the full spectrum of variants in precision oncology.

## Conclusion

The field of SV detection is continuously improving through advancements in sequencing technologies and tools. These advancements will contribute to discoveries into the role of SVs in cancer, as well as the incorporation of SVs in precision oncology programs. Nevertheless, SV detection and interpretation in tumor samples is complicated by unique biological and technical challenges, i.e., contamination, intra-tumor heterogeneity and aneuploidy. These challenges are addressed by algorithms specialized in identifying TSSVs from tumor-normal paired sequencing data, which requires both SV detection and distinguishing tumor-specific variants.

Based on studies of normal genomic variation, a multi-platform approach is necessary to detect the full spectrum of variants and reduce false positives. Truth sets and procedures developed for SV detection from short-read data show that combining multiple tools improves precision and recall. Despite this, short-read sequencing has inherent limitations such as GC coverage bias and mapping ambiguities leading to inaccessible genomic regions. Long-read sequencing technologies can resolve large, complex SVs and improve coverage, but have lower per-nucleotide accuracy, higher costs and sample requirements. SV detection tools for long-read data have yet to mature with performance assessments and truth sets lacking.

Integration of long-read and short-read data is likely required for complete characterization of tumor genomes. However, adopting sequencing technologies in clinical laboratories requires a clear added value compared to the standardized assays, as well as being fast and affordable. Considering IL and 10× provide high accuracy WGS at low sample requirements, they are most feasible for tumor-normal sequencing in a clinical setting. Supplementary low-coverage sequencing with ONT can cover regions inaccessible to short-read WGS and aid in variant phasing. Alternatively, RNA sequencing has proven to be highly beneficial in a clinical setting for the detection of gene fusion events.

In conclusion, improving detection of TSSVs by integrating data derived from multiple platforms and detection tools enables the use of TSSVs in precision oncology and research into their role in cancer. With accurate TSSV datasets becoming more available, previously unchartered territories of variant types can be explored to potentially discover novel SV cancer driver events.

## Data Availability

No datasets were generated or analyzed during this study.
